# Growth restriction in gastroschisis: quantification of its severity and exploration of a placental cause

**DOI:** 10.1186/1471-2431-11-90

**Published:** 2011-10-17

**Authors:** Nathaniel R Payne, Susan C Simonton, Sam Olsen, Mark A Arnesen, Kathleen M Pfleghaar

**Affiliations:** 1 Department of Epidemiology, Michigan State University, B601 West Fee Hall, East Lansing, Michigan 48824, USA; 2 Department of Quality and Safety, Children's Hospitals and Clinics of Minnesota, 2525 Chicago Avenue South, Minneapolis, MN 55404, USA; 3 Department of Pathology, Children's Hospitals and Clinics of Minnesota, 2525 Chicago Avenue South, Minneapolis, MN 55404, USA; 4 Department of Neonatology, Children's Hospitals and Clinics of Minnesota, 2525 Chicago Avenue South, Minneapolis, MN 55404, USA; 5 Department of Pathology, Abbott Northwestern Hospital, Minneapolis, MN 55407, USA; 6 Department of Perinatology, Abbott Northwestern Hospital, Minneapolis, MN 55407, USA

## Abstract

**Background:**

Gastroschisis patients are commonly small for gestational age (SGA, birth weight [BW] < 10^th ^centile). However, the extent, symmetry and causes of that growth restriction remain controversial.

**Methods:**

We compared BW, crown-heel length (LT), occipitofrontal circumference (OFC) and ponderal index (PI) in 179 gastroschisis cases and 895 matched controls by univariate and multiple regression. Fetal ultrasounds (N = 80) were reviewed to determine onset of growth restriction. Placental histology was examined in 31 gastroschisis patients whose placental tissue was available and in 29 controls.

**Results:**

Gastroschisis cases weighed less than controls (BW = 2400 ± 502 g vs. 2750 ± 532 g, p < 0.001) and their BW frequency curve was shifted to the left, indicating lower BW as a group compared to controls (p < 0.001 by Kolmogorov-Smirnov test). BW differences varied from -148 g at 33 weeks to -616 g at 38 weeks gestation. Intrauterine growth restriction was symmetric with gastroschisis patients having a shorter LT (45.7 ± 3.3 vs. 48.4 ± 2.7 cm, p < 0.001), smaller OFC (31.9 ± 1.9 vs. 32.9 ± 1.6 cm, p < 0.001), but larger ponderal index (2.51 ± 0.37 vs. 2.40 ± 0.16, p < 0.001) compared to controls. Gastroschisis patients had a similar reduction in BW (-312 g, 95% confidence interval [CI] = -367, -258) compared to those with chromosomal abnormalities (-239 g, CI = -292, -187). Growth deficits appeared early in the second trimester and worsened as gestation increased. Placental chorangiosis was more common in gastroschisis patients than controls, even after removing all SGA patients (77% vs. 42%, p = 0.02).

**Conclusions:**

Marked, relatively symmetric intrauterine growth restriction is an intrinsic part of gastroschisis. It begins early in the second trimester, and is associated with placental chorangiosis.

## Background

Gastroschisis is a unique congenital anomaly appearing as a defect in the abdominal wall usually to the right of the umbilicus. It commands increasing interest because of its rising prevalence [1-4] and clinical impact [5,6]. Most gastroschisis cases present with an isolated anomaly, which develops around the 6^th ^gestational week [7] and is not usually associated with chromosomal abnormalities [8,9]. However, almost all studies report gastroschisis patients have an increased risk of being small for gestational age (SGA, birth weight [BW] < 10^th ^centile) [6,10-13]. Previous fetal studies reported not only a high prevalence of SGA (up to 61%), but also a leftward shift in the BW distribution compared to intrauterine fetal growth curves [11,13]. Determining the appropriate comparison standard for gastroschisis cases may not be straightforward, since these patients' mothers have a unique demographic profile that may differ from that of the population from which the standards were developed [14-19]. Mothers of gastroschisis patients are more likely to be young, primigravida, undernourished, smokers, and tend to have a low BMI, [2,15-19] all factors that are also associated with decreased intrauterine growth [20] and which might confound any association of gastroschisis with intrauterine growth. The extent, symmetry and causes of growth restriction remain controversial. The purpose of this study was to characterize and quantitate the fetal growth restriction in gastroschisis and to explore the role of placental dysfunction as a cause of this growth deficit.

## Methods

### Study design

**Figure 1:**
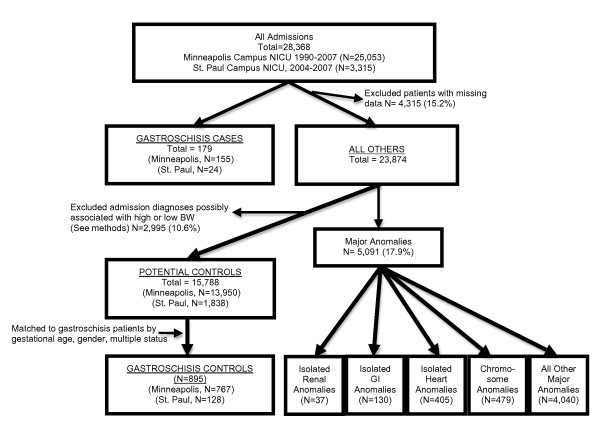
**Flow diagram showing the total study population, exclusions, and final sample sizes**. Percentages are calculated using the total number of available patients before exclusions.

This retrospective, case-control study was a secondary analysis of data collected on all newborns admitted to the NICUs of Children's Hospitals and Clinics of Minnesota. We conducted four analyses in this study. Analysis #1) We compared BW, crown-heel length (LT), occipitofrontal circumference (OFC), and ponderal index (PI) at birth in gastroschisis cases to that in matched controls without major congenital anomalies and to neonatal and fetal growth standards [21-24]. Analysis #2) We examined the same measures of size at birth comparing gastroschisis patients to other groups of anomalies: a) isolated gastrointestinal (GI) anomalies other than gastroschisis, b) isolated renal anomalies, c) isolated cardiac anomalies, d) chromosomal anomalies, e) all other anomalies, and f) those with no recorded, major anomaly (Figure 1). Analysis #3) We examined estimated fetal weight determined by prenatal ultrasound in gastroschisis patients. Analysis #4) Finally, we examined placental findings in gastroschisis patients and controls.

### Subjects

Our study population was live-born patients with gastroschisis. The study sample included all gastroschisis cases admitted to Children's Hospitals and Clinics of Minnesota either at the Minneapolis campus from 1 January 1990 to 31 December 2007 or at the St. Paul campus from 1 January 2004 to 31 December 2007 (Figure 1). We identified 179 cases of gastroschisis. There were no delivery room deaths among gastroschisis patients. Information on terminations, stillbirths, and fetal deaths was not available. The Institutional Review Boards of Children's Hospitals and Clinics of Minnesota (No. 0811-104) and Abbott Northwestern Hospital (No. 2558-1E) approved this study.

For the first analysis, we matched five controls to each case on a case-to-case basis by gestational age, gender, and multiple gestation status, using computerized random sampling of potential controls without replacement (Table 1). Since half of all gastroschisis patients are born at ≤36 weeks gestation and virtually all infants with gestational age ≤36 weeks gestation are admitted to a NICU, controls came from the same NICU population as cases (N = 28,368, Figure 1). We excluded potential controls with missing data (N = 4315, 15.2%) or major anomalies (N = 5091, 17.9%) (Figure 1). For the purposes of this study, the following recorded diagnoses and procedures represented or potentially represented major anomalies: renal malformations, congenital heart disease (excluding patent ductus arteriosus), central nervous system malformations, chromosome abnormalities, major skeletal anomalies, recognizable dysmorphic syndromes and thoracic, abdominal, head/neck, or heart surgery. No congenital infections were diagnosed in cases or controls. To minimize the risk of bias in controls' growth measures, [25,26] we included only patients with admission diagnoses unlikely to be associated with abnormal intrauterine growth: prematurity, respiratory distress, R/O sepsis, unstable temperature, and "observation status."

**Table 1:** Characteristics of study cases and controls

Feature	Gastroschisis (n = 179) number (%)	Controls (n = 895) number (%)	**p-value**^**a**^
Gestational age (weeks) (mean ± sd)	36 ± 2	36 ± 2	1.000
Maternal age (years) (mean ± sd)	22 ± 4	30 ± 3	<0.001
Age ≤ 20 years (%)	69 (38.6)	44 (5.0)	
Age 20-24 years (%)	70 (39.1)	154 (17.2)	
Age 25-29 years (%)	31 (17.3)	248 (27.7)	
Age ≥ 30 years (%)	9 (5.0)	449 (50.2)	
Maternal race			<0.001
Caucasian (%)	140 (78.2)	741 (82.8)	
African American (%)	7 (3.9)	90 (10.1)	
Asian (%)	19 (10.6)	38 (4.3)	
Hispanic (%)	8 (4.5)	20 (2.2)	
Native American (%)	5 (2.8)	6 (0.7)	
Female	84 (47.0)	420 (47.0)	1.000
Inborn (%)	155 (86.6)	697 (77.9)	0.008
Birth weight <10^th ^centile^b ^(%)	44 (24.6)	69 (7.7)	<0.001
Gestational hypertension/pre-eclampsia (%)	0	148 (16.5)	N/A^e^
Single (%)	113 (63.1)	224 (25.0)	<0.001
Minor anomalies (%)	24 (13.4)	39 (4.4)	<0.001
Multiple gestation	4 (2.2)	20 (2.2)	1.000
Primigravida	110 (61.5)	285 (31.8)	<0.001
Gestational diabetes^f^	1 (0.6)	63 (7.3)	0.010
Pre-existing (before pregnancy) diabetes^f^	0	27 (3.3)	N/A^e^
Maternal smoking	52 (29.1)	138 (15.4)	<0.001
Maternal illicit drug use	7 (3.9)	32 (3.6)	0.829
Maternal alcohol use	7 (3.9)	16 (1.8)	0.082
Center (Minneapolis)	155 (86.6)	767 (85.7)	0.754
Era (1999-2007)	129 (72.1)	581 (64.9)	0.065
Birth weight (g)	2400 ± 502	2750 ± 532	<0.001
Birth weight z-score	-0.65 ± 0.86	0.11 ± 0.49	<0.001
Crown-heel length (cm)	45.7 ± 3.3	48.4 ± 2.7	<0.001
Crown-heel length z-score^c,d^	-0.50 ± 1.19	0.68 ± 0.55	<0.001
Occipitofrontal circumference (OFC)	31.9 ± 1.9	32.9 ± 1.6	<0.001
OFC z-score^d ^(cm)	-0.36 ± 0.87	0.24 ± 0.45	<0.001
Ponderal index	2.51 ± 0.37	2.40 ± 0.16	<0.001
Ponderal index z-score	-0.06 ± 0.85	-0.30 ± 0.34	<0.001

^a ^Determined for continuous variables using paired t-test comparing the gastroschisis cases as individuals and the mean of the five matched controls' values as the paired control. Determined for the selected dichotomous and categorical variables using conditional logistic regression with adjustment for matching.

^b ^Small for gestational age status determined as BW < 10^th ^centile for gestational age using the standards of Fenton, et al.[21]

^c ^Z-scores calculated by method of Cole, et al.[22]

^d ^Length and OFC were missing in two gastroschisis cases, N = 177 for paired t-tests.

^e ^Unable to estimate p-value with no cases in one or more cells.

^f ^Presence or absence of pre-existing and gestational diabetes missing for 12 cases and 11 controls.

For the 2^nd ^analysis, we examined additional patient groups with isolated renal (N = 37), GI anomalies other than gastroschisis (N = 130), cardiac (N = 405), chromosomal (N = 479) and any other congenital anomaly (N = 4040, Figure 1). Isolated renal anomalies included 26 cases with hydronephrosis and 11 cases of multicystic/polycystic kidneys. Isolated GI anomalies included imperforate anus- 52 cases, Hirschsprung's Disease- 47, and intestinal atresia- 31. The three most commonly recorded cardiac diagnoses were d-transposition of the great arteries- 114, hypoplastic left heart syndrome- 74, and aortic coarctation- 63 cases. The three most common chromosomal anomalies were trisomy 21- 225, trisomy 18- 31, and trisomy 13- 16 cases. All remaining patients with major anomalies were included in the final group of "any other major anomaly". These four groups of major anomalies and the gastroschisis cases were compared to the 15,788 patients without major anomalies (Figure 1).

For the 3^rd ^analysis using prenatal ultrasound data, there were 80 women with at least one available ultrasound evaluation. For the 4^th ^analysis, there were 31 gastroschisis and 29 control patients with available placental tissue.

### Neonatal clinical data

Data were collected concurrent with hospitalization as part of an ongoing NICU outcomes monitoring project. BW, LT, and OFC were obtained by NICU nurses and neonatal nurse practitioners. Prenatal ultrasounds were interpreted by board-certified perinatologists. Fetal weight estimates were based on the standards of Hadlock, et al [14]. A board-certified pathologist (MAA) or pediatric pathologist (SCS) reviewed placental histological findings. Placental weight was obtained after draining, trimming, and patting the placenta dry. The placental weight z-score was calculated using published standards [27,28].

### Clinical definitions

Gestational age came from obstetrical estimates based on last menstrual period and corrected by early second trimester ultrasound, if available. If physical exam indicated a gestational age > 2 weeks different from the obstetrical estimate, the estimate from the physical exam was used. Small for gestational age (SGA) was defined as BW < 10^th ^centile [21]. Ponderal index (PI), average neonatal weight gain and fetal growth were assessed using published standards [24,28,29]. Ponderal index was defined as weight in kg divided by length in meters cubed [23]. Gestational hypertension and pre-eclampsia were determined by the treating obstetrician's assessment and collapsed into a single group, gestational hypertension. Maternal smoking, a dichotomous variable, was determined by the mother's report as present if the mother smoked after she knew she was pregnant.

### Placental analysis

We examined the original slides of available placentas to determine the presence of abnormalities. All slides were examined by a board-certified pediatric pathologist (SCS). The diagnosis of chorangiosis, capillary proliferation in placental terminal villi, was made using the definition of Altschuler and Baergen [30,31] with slight modication as follows: 1) Focal chorangiosis was defined as > 10 capillaries in > 10 terminal villi in 10 fields at 10× magnification in each of 1-2 of 3 slides. Diffuse chorangiosis was defined as > 10 capillaries in > 10 terminal villi in 10 fields at 10X magnification in each of 3 slides. Chorangiosis usually represents chronic hypoxic environment for the fetus and manifests an attempt to enlarge the placental diffusional surface [30,31].

### Statistical analysis

For the 1^st ^analysis, we compared cases with matched controls using univariate conditional logistic regression for dichotomous and categorical variables. Continuous variables were compared by the paired t-test after averaging values for the five controls, thus consolidating the five controls' values into a single value. BW distributions of cases and controls were analyzed using the Kolmorogov-Smirnov test. We adjusted for possible covariates using multiple linear regression. We included in the regresion equation all available variables that were associated with BW by univariate analysis with a p-value < 0.10 or that might reasonably influence BW [20]. Substantial collinearity was not present (variance inflation factors, 1.02 - 1.71). Gestational age, gender, and multiple gestation accounted for 50% of the variation in BW. These variables were not included in the regression analysis because they were perfectly matched among cases and controls. The regression equation without these variables explained 10% of the variation in BW and LT, 9% of the variation in OFC and 7% in the variation of PI. Our study had >99% power to detect a ≥10% difference in the mean BW of gastroschisis patients and controls at the p = 0.05 level. For analysis #2, we also used multiple regression to compare the different groups with anomalies to the group without anomalies. We included the same covariates as above and added gender, gestational age and multiple gestation, since these data were not matched. Variance inflation factors were 1.01 - 1.13. Regression results accounted for 82%, 81%, 78% and 10% of the variation in BW, LT, OFC, and PI respectively. For analysis #3, we used univariate descriptive statistics and sign ranks tests to compare prenatal ultrasound estimates of fetal weight and birth weight. For analysis #4, we used the Fisher exact test to compare placental findings in cases and controls. We used only 2-sided p-values and made no adjustment for multiple comparisons. All analyses were performed with Stata, version 11.1 (College Park, TX).

## Results

### Patient population and demographics

**Figure 2:**
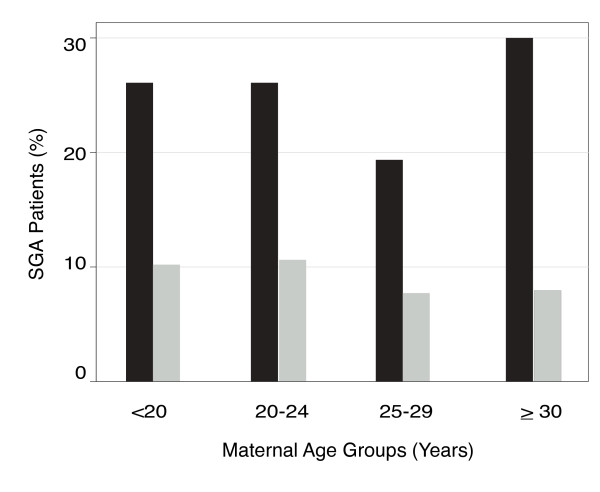
**The percent of SGA infants (birth weight <10**^**th **^**centile) by maternal age group in gastroschisis cases (black bars) and controls (gray bars)**. For neither gastroschisis nor controls was the rate of SGA significantly different among the maternal age groups (p = 0.931 and p = 0.326, respectively by Fisher exact test).

We identified 179 gastroschisis cases to whom we matched 895 controls (5:1 ratio, Figure 1) with the following admission diagnoses: respiratory distress (N = 390), prematurity (N = 353), rule out sepsis (N = 67), need for observation (N = 72), and unstable temperature (N = 13). Mothers of gastroschisis cases were significantly younger, more commonly single, Asian, primigravid, and less likely to have gestational hypertension compared to mothers of controls (Table 1). Gastroschisis cases were more likely to be inborn, SGA and have minor anomalies (in addition to gastroschisis) compared to controls (Table 1). Examples of these relatively minor anomalies were supernumerary digit, talipes equinovarus deformity, and cyst in filum terminale. SGA was more common among gastroschisis patients across all maternal age groups (Figure 2).

### #1 Analysis: Case-control comparison

**Figure 3:**
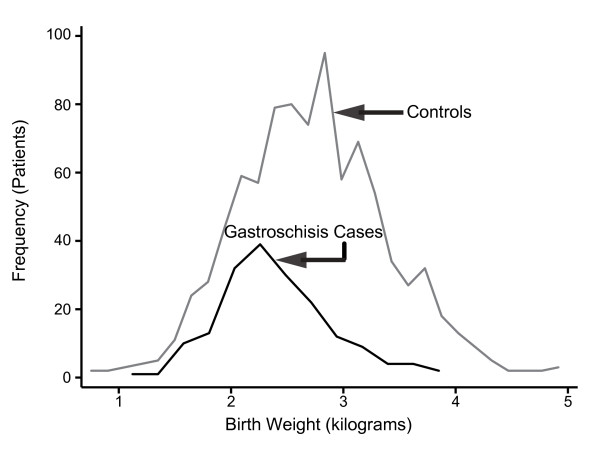
**Frequency line graphs of the birth weight distribution of gastroschisis cases (black line) and controls (gray line)**. Kolmogorov-Smirnov test confirmed that this difference was significant (p < 0.001).

**Figure 4:**
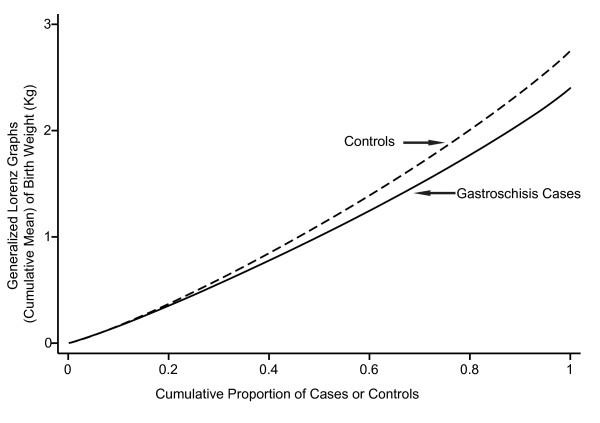
**Generalized Lorenz graphs of the birth weight for gastroschisis cases (black line) and controls (interrupted gray line)**. The X-axis represents the proportion of the population of both cases and controls. The Y-axis represents the cumulative mean birth weight, calculated as cumulative birth weight at the given proportion of the population divided by the total population. These curves are a measure birth weight distribution and indicate gastroschisis cases have a lower cumulative mean birth weight at almost all proportions of the study sample.

Mean BW of gastroschisis cases was 350 grams less than controls (Mean ± standard deviation [sd]), 2400 ± 502 vs. 2750 ± 532 grams, p < 0.001). The BW frequency curve for gastroschisis cases was shifted to the left compared to controls, suggesting that gastroschisis cases overall had significantly lower BW than controls (Figure 3, Kolmogorov-Smirnov test, p < 0.001). Generalized Lorenz graphs of the cumulative mean birth weight also demonstrated this difference (Figure 4). Similarly, mean length was 2.7 cm shorter (LT 45.7 ± 3.3 vs. 48.4 ± 2.7 cm, p < 0.001) and mean OFC was 1.0 cm smaller (31.9 ± 1.9 vs. 32.9 ± 1.6 cm, p < 0.001) than controls (Table 1). The PI was slightly higher than that of controls (2.51 vs. 2.40, p < 0.001). These findings were consistent with relatively symmetric growth restriction.

Since the gastroschisis babies who were severely growth restricted, BW < 10^th ^centile, might skew the analysis, we repeated the comparisons after eliminating the 44 SGA cases and their associated controls. Gastroschisis cases were still lighter by 199 g (2530 ± 489 vs. 2729 ± 563 g, p < 0.001), shorter by 1.9 cm (46.4 ± 3.1 vs. 48.3 ± 2.8 cm, p < 0.001), and had a smaller OFC by 0.6 cm (32.2 ± 1.9 vs. 32.8 ± 1.7 cm., p < 0.001) compared to controls. Similarly, PI was again very slightly higher in cases than controls (2.5 ± 0.4 vs. 2.4 ± 0.2, p < 0.001). Growth restriction occurred across the BW distribution of gastroschisis patients whether or not they met the technical definition of SGA (Figure 3).

**Figure 5:**
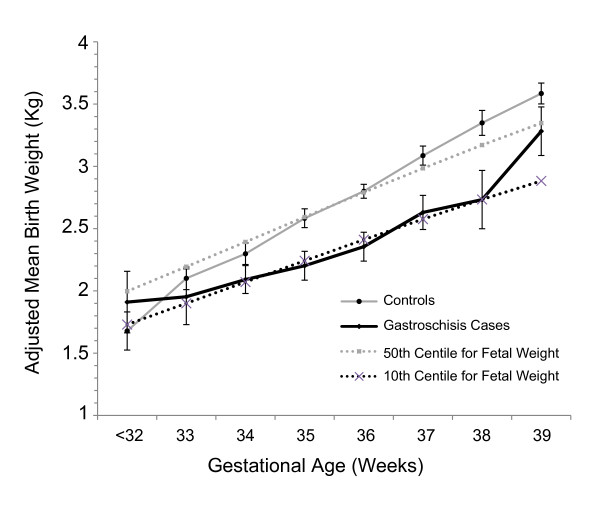
**Adjusted mean BW of gastroschisis cases (black line) and controls (gray line) by gestational age with the 95% confidence intervals represented by the error bars**. The 10^th ^and 50^th ^centiles were obtained from published standards[24]. BW was adjusted using the regression equation from Table 2 and included maternal race, cigarette smoking, GHP, recreational drug use, previous pregnancies and inborn status. From 33 weeks to about 38 weeks gestation, gastroschisis cases weighed progressively less than controls, changing from -148 grams to -616 grams. The percentage of BW deficit in gastroschisis cases increased from 7% at 33 weeks gestation to 18% at 38 weeks. The small number of patients available for analysis prior to 33 and after 38 weeks resulted in wide confidence intervals and potentially unreliable estimates.

We then adjusted for other demographic features that might confound the association of poor fetal growth and gastroschisis. Black race, Asian race, maternal hypertension, illicit drug use and nulliparity were all negatively associated with BW (Table 2). Maternal diabetes was positively associated with BW. After adjustment for potential confounders, there remained a 317 g reduction in BW (95%CI = -415, -218, p < 0.001), a 2.5 cm reduction in LT (95% CI = -3.1, -1.9, p < 0.001), 0.8 cm reduction in OFC (95% CI = -1.1, -0.5), and a similar increase in PI of 0.1 (95% CI = 0.03, 0.17, p = 0.006) with gastroschisis compared to controls (Table 2). There was an interaction between gastroschisis and gestational age. From 33 to 38 weeks gestation, the BW difference between cases and controls increased from -148 g to -616 g (7% to 18% of controls' BW, Figure 5). Therefore, gastroschisis patients became progressively lighter compared to controls of the same gestational age.

**Table 2:** Unadjusted and adjusted analysis of birth weight among gastroschisis patients and Controls

		Unadjusted			Adjusted	
	
	**Coefficient**^**a**^	**95% CI**^**b**^	p-value	**Coefficient**^**c**^	**95% CI**^**b**^	p-value
Gastroschisis	-350	-415, -286	<0.001	-317	-415, -218	<0.001
Gestational age (weeks)	228	213, 244	<0.001	ND^c^	ND	ND
Male gender	107	-39, 253	0.151	ND^c^	ND	ND
Multiple gestation	-241	-391, -92	0.002	ND^c^	ND	ND
Maternal race/ethnicity						
White	Referent			Referent		
African American	-250	-363, -136	<0.001	-236	-368, -104	0.001
Native American	1	-429, 431	0.996	277	-181, 735	0.235
Asian	-219	-344, -95	0.001	-178	-301, -55	0.005
Hispanic	-182	-480, 115	0.229	-178	-510, 155	0.292
Gestational diabetes	134	-53, 323	0.160	77	-106, 260	0.407
Pre-existing diabetes	628	348, 908	<0.001	689	406, 973	<0.001
Maternal smoking	-220	-313, -128	<0.001	-142	-249, -34	0.010
Maternal hypertension	-251	-372, -129	<0.001	-354	-473, -234	<0.001
Maternal illicit drug use	-378	-545, -211	<0.001	-255	-453, -58	0.012
Maternal alcohol use	-390	-603, -176	0.005	-143	-358, 71	0.189
Single mother	-234	-316, -152	<0.001	-59	-161, 42	0.250
Maternal age (years)	13	7, 18	<0.001	-2	-9, 6	0.654
Nullipara	-164	-246, -82	<0.001	-98	-192, -6	0.037
Outborn (yes = 1, no = 0)	239	141, 337	<0.001	136	42, 230	0.005
Era (1999-2007)	1	-72, 74	0.980	27	-44, 98	0.452

^a ^Obtained by univariate regression

^b ^CI= 95% confidence interval

^c ^Adjusted coefficient obtained by multiple regression. Since cases and controls were perfectly matched for gestational age, gender, and multiple gestation, these variables were not included in the multiple regression analyses. All other potential covariates were included (see methods).

Slow neonatal growth exacerbated slow intrauterine growth. Mean weight gain for gastroschisis cases was 6.5 g/kg/day. Gastroschisis cases dropped from a mean BW z-score of -0.65 ± 0.86 (31st centile) at birth to -0.94 ± 0.79 (23rd centile) at discharge. Although cross-sectional birth weight standards are not designed to measure longitudinal growth, our findings suggested that postnatal growth deficits compounded those occurring before birth in gastroschisis cases.

### #2 Analysis: Comparison of gastroschisis and other patients with anomalies

We then examined intrauterine growth restriction in gastroschisis cases compared to other patients with major anomalies (Table 3). Gastroschisis, chromosomal anomalies and isolated congenital heart disease were all associated with significantly lower BW when compared to those without major anomalies. After adjusting for covariates by multiple regression analysis, gastroschisis was associated with a 312 g (CI 368, 259; p < 0.001) reduction in BW compared to infants with no major anomalies. The BW reduction associated with gastroschisis was somewhat larger than that seen with chromosomal anomalies (234-g reduction, CI -285, -182; p < 0.001 grams). LT and OFC showed similar differences before (Table 3) and after adjustment for covariates (data not shown). Gastroschisis was unique among the groups of anomalies that we examined in causing severe growth restriction, similar in degree to that seen with chromosomal abnormalities.

**Table 3:** Birth weight, length, and occipitofrontal circumference among NICU admissions with and without anomalies

	Gastroschisis (N = 179)	No major anomaly (N = 15,788)	Renal anomalies (N = 37)	Other GI anomalies (N = 130)	Chromosome abnormalities (N = 479)	Congenital heart disease (N = 405)	Any other major anomalies (N = 4,040)
Gestational age (wks)	35.7 ± 2.0	34.2 ± 4.0	34.1 ± 5.1	38.3 ± 2.7	36.5 ± 3.3	38.5 ± 2.2	33.7 ± 5.7
Unadjusted BW (g)	2400 ± 502	2387 ± 918	2500 ± 1.140	3296 ± 701	2660 ± 861	3216 ± 638	2331 ± 118
BW z-score	-0.64 ± 0.86	0.01 ± 0.93	0.25 ± 1.00	0.13 ± 0.96	-0.44 ± 1.3	-0.11 ± 0.98	-0.04 ± 1.14
BW coefficient^a^	-310 (-366, -254)^b^	Referent	45 (-93, 184)	26 (-55, 107)	-244 (-298, -192) ^b^	-94 (-141, -47) ^b^	-7 (-23, 10)
Unadjusted LT (cm)	45.7 ± 3.3	46.0 ± 5.7	44.8 ± 6.8	50.6 ± 3.6	46.7 ± 5.3	50.4 ± 3.7	44.3 ± 8.1
LT z-score	-0.50 ± 1.19	0.42 ± 1.15	0.05 ± 1.69	0.57 ± 1.04	-0.37 ± 1.53	0.37 ± 1.24	0.11 ± 1.41
LT coefficient^a^	-2.1 (-2.5, -1.7)^b^	Referent	-1.3 (-2.6, -0.1)^d^	-0.7 (-1.1, -0.2)^c^	-2.3 (-2.7, -2.0)^b^	-1.2 (-1.5, -0.9)^b^	-1.1 (-1.2, -1.0)^c^
Unadjusted OFC (cm)	31.9 ± 1.9	31.5 ± 3.5	31.0 ± 4.4	34.2 ± 2.3	31.9 ± 3.1	33.9 ± 1.9	30.7 ± 5.3
OFC z-score	-0.36 ± 0.86	0.16 ± 1.02	0.04 ± 1.01	0.04 ± 0.97	-0.56 ± 1.30	-0.24 ± 0.95	0.15 ± 1.52
OFC coefficient^a^	-0.5 (-0.8, -0.3)^b^	referent	-0.6 (-1.1, 0.1)^d^	-0.6 (-0.8, -0.3)^b^	-1.3 (-1.5, -1.2)^b^	-1.0 (-1.2, -0.9)^b^	-0.3 (-0.4, -0.2)^b^
Ponderal index	2.5 ± 0.37	2.3 ± 0.48	2.6 ± 0.89	2.5 ± 0.33	2.5 ± 0.49	2.5 ± 0.34	2.4 ± 0.53
PI z-score	-0.06 ± 0.85	-0.24 ± 0.91	0.32 ± 1.86	-0.25 ± 0.53	-0.02 ± 1.18	-0.24 ± 0.59	-0.02 ± 1.17
PI coefficient^a^	0.10 (0.04, 0.15)^b^	referent	0.27 (0.00, 0.53)^d^	0.01 (-0.04, 0.03)	0.10 (0.05, 0.15)^b^	-0.01 (-0.04, 0.03)	0.08 (0.06, 0.10)^b^

^a ^Determined using covariates gestational age, gender, multiple gestation status, maternal race, maternal cigarette smoking, GHP, pre-existing and gestational diabetes, maternal illicit drug use, previous pregnancies, and inborn status. The regression model explained 82% of the variation in birth weight (R^2 ^= 0.818), 81% of the variation in LT (R^2 ^= 0.810), 78% of the variation in OFC (R^2 ^= 0.777) and 10% of the variation of PI (R^2 ^= 0.103).

^b ^p < 0.001

^c ^p < 0.01

^d ^p < 0.05

### #3 Analysis: Onset of decreased intrauterine growth

We reviewed the prenatal ultrasounds of 80 women pregnant with a baby with gastroschisis. Their first ultrasound was obtained at a median of 26 weeks (range 21 - 38) gestation. At the first ultrasound, almost all gastroschisis patients had a low estimated fetal weight (median estimated fetal weight centile = 27, range 3 - 70). Among those with more than one fetal ultrasound, 32/54 (59%) had either a drop or no change in estimated fetal weight centile between the first and last fetal weight estimate. The mean (±sd) time between the first and last fetal ultrasound was 9 ± 3 weeks. Fetal growth restriction appeared by at least the second trimester and generally worsened as gestation advanced.

We then compared estimated fetal weight percentile and birth weight percentile in the 61 women who had an ultrasound within 3 weeks of delivery (Table 4). Estimated fetal weight was lower than actual birth weight, but this was probably attributable to the interval between the last ultrasound measurement and delivery. Estimated fetal weight percentile and measured birth weight percentile did not differ (Table 4).

**Table 4:** Comparison of Estimated Fetal Weight by Ultrasound and Measured Birth Weight

	**Last ultrasound measurement (N = 61)**^**a**^	**Measurement at birth (N = 61)**^**a**^	**p-value**^**b**^
Weight (grams) median ( IQR^c^)	1956 (1607-2273)	2300 (2050-2660)	<0.001
Weight percentile median (IQR)	27 (8-38)	28 (10-42)	0.221
Gestational age (weeks) Median (IQR)	34 (33-36)	36 (34-37)	<0.001

^a ^There were 61 women who had an ultrasound exam at ≤ 3 weeks before delivery

^b ^P-value determined by signed-rank test. The data were skewed and therefore non-parametric tests were used for this small data set.

^c ^Interquartile range.

### #4 Analysis: Placental abnormalities associated with gastrochisis cases

**Figure 6:**
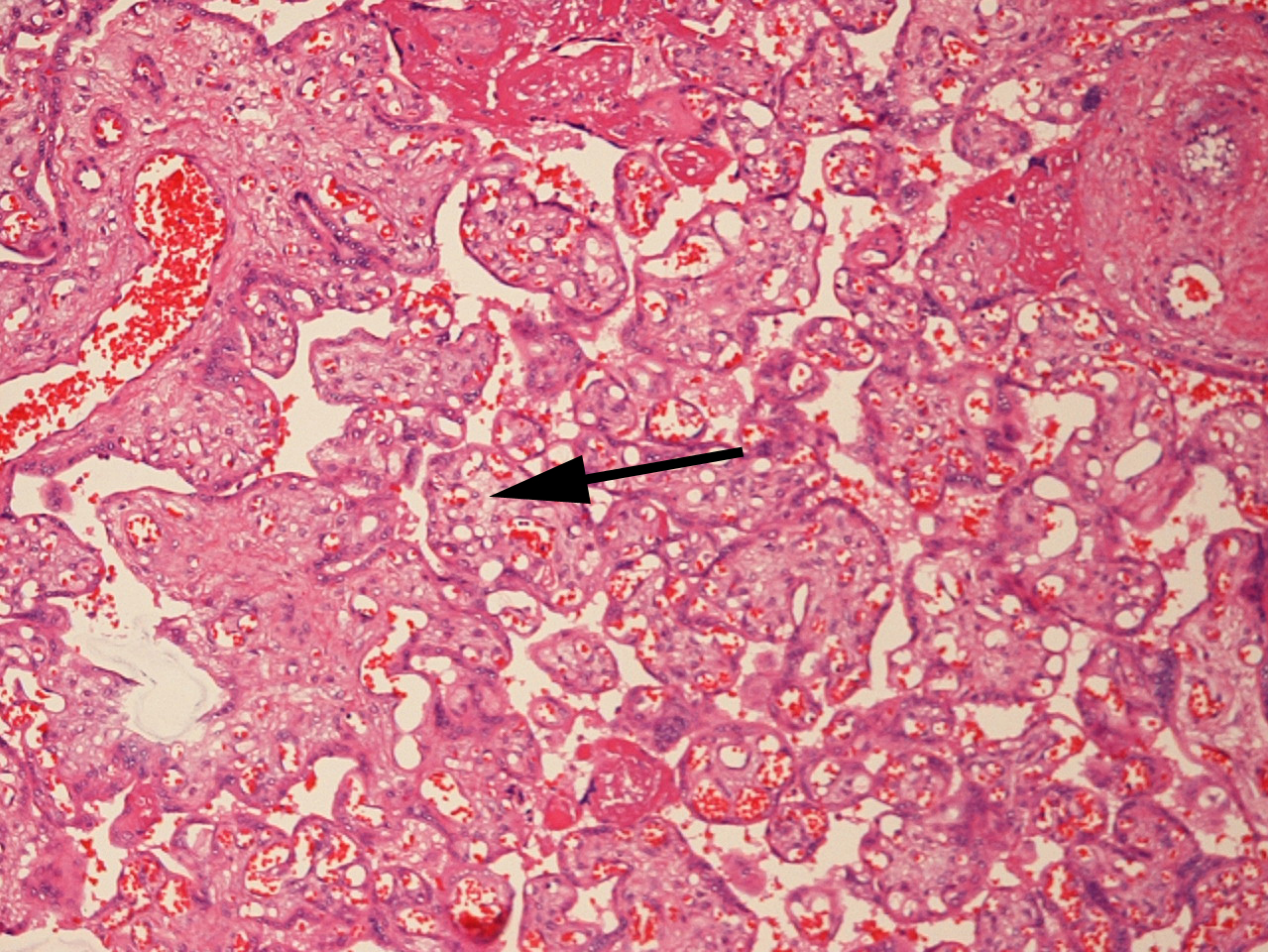
**Figure 6. A 10× photomicrograph of chorangiosis in a placenta from a woman who delivered a patient with gastroschisis**. The arrow points to an area with multiple vascular channels. Diffuse chorangiosis was defined as ≥ 10 capillaries in ≥ 10 terminal villi in 10 fields at 10× magnification in each of 3 areas (slides). Red blood cells can be seen in many of the capillaries. Capillary proliferation can be seen in numerous terminal villi.

**Figure 7:**
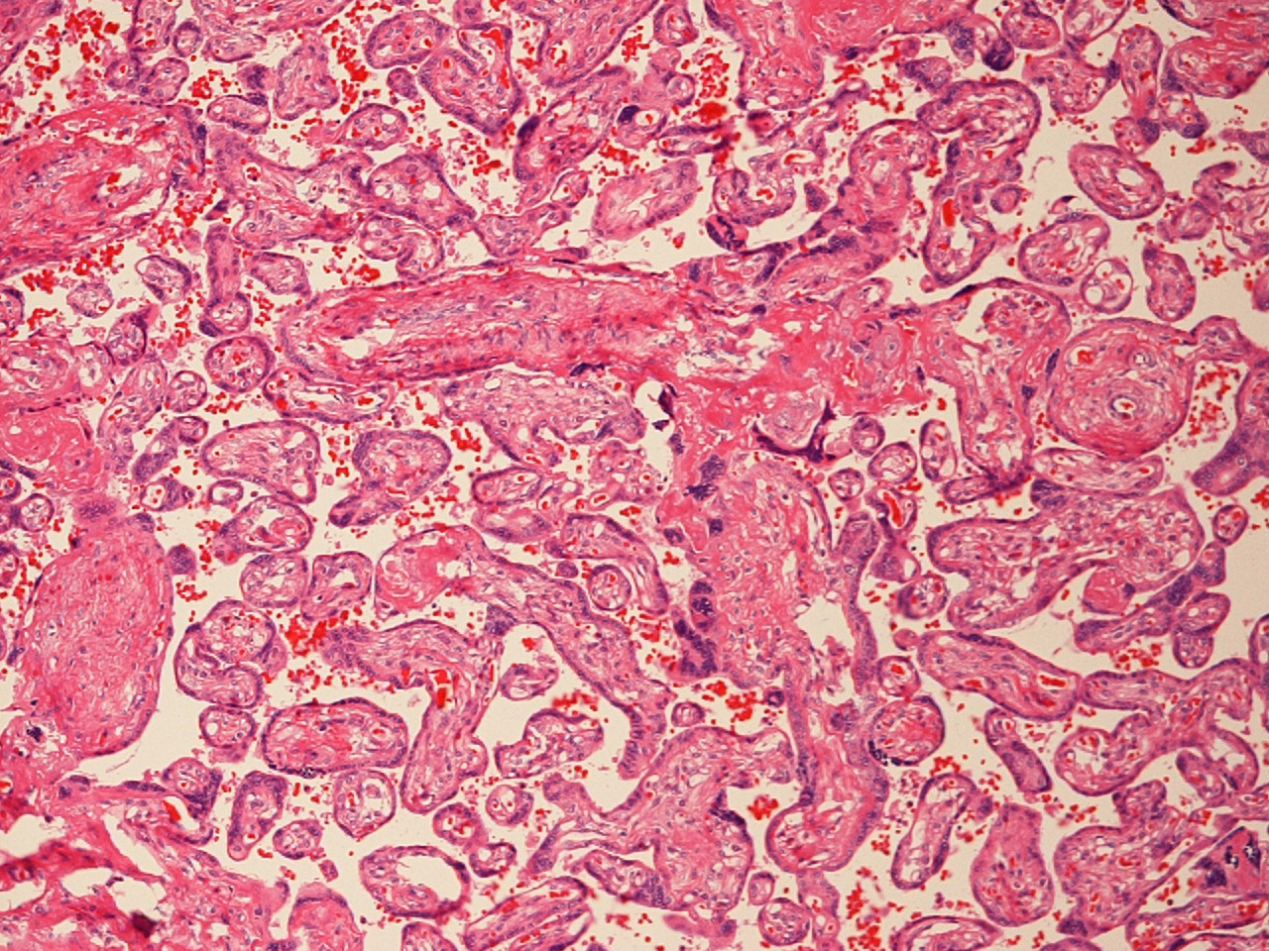
**Figure 7. A 10× photomicrograph of a placenta from a woman who delivered a control patient**. Chorangiosis is not present in this photmicrograph.

We examined the placental weight, placental weight z-scores and histology in the 31 available placentas from gastroschisis cases and from 29 controls. There was no significant relationship between placental wt z-score and BW z-score (R = 0.204, p = 0.063). However, placentas of gastroschisis cases had a significantly higher prevalence of chorangiosis (81% vs. 41%, p = 0.003) and villous edema (33% vs. 0%, p = 0.005), but not chorioamnionitis (42% vs. 48%, p = 0.796, Table 5). Even after removing SGA patients (chorangiosis is associated with SGA), placentas from gastroschisis patients still had chorangiosis more frequently than controls (77% vs. 42%, p = 0.02). Representative photomicrographs of chorangiosis in the placenta associated with a gastroschisis case and no chorangiosis in a control appear in Figures 6 and 7, respectively. Among patients with chorangiosis, only one (a gastroschisis case) infant's mother had diabetes, a condition also known to be associated with chorangiosis. No other significant findings were seen, such as infarcts or fibrinoid deposition.

**Table 5:** Placental findings associated with gastroschisis

Placental findings	Controls N = 29	Gastroschisis N = 31	p-value
Placental weight z-score (mean ± sd)^a^	0.24 ± 1.29	-0.03 ± 1.07	0.804^b^
Chorioamnionitis (%)	14 (48)	13 (42)	0.796^c^
Any chorangiosis (%)^d^	12 (41)	25 (81)	0.003^c^
Focal chorangiosis (%)^d^	8 (28)	12 (39)	0.419^c^
Diffuse chorangiosis (%)^e^	4 (14)	13 (42)	0.022^c^

^a ^Excludes the placentas of patients who were SGA (n = 9), had hydrops (n = 5), or both (n = 3).

^b ^Rank-sum test

^c ^Fisher exact test

^d ^Focal chorangiosis was defined as ≥ 10 capillaries in ≥ 10 terminal villi in 10 fields at 10× magnification in each of 1-2 areas (slides).

^e ^Diffuse chorangiosis was defined as ≥ 10 capillaries in ≥ 10 terminal villi in 10 fields at 10× magnification in each of 3 areas (slides).

## Discussion

Our study further characterizes the association of poor fetal weight gain and gastroschisis that has been reported by others [5,6,10-13,32-34]. Intrauterine growth restriction of some degree often accompanies gastroschisis, even when the BW percentile is > 10^th ^percentile [11]. In many cases, the degree of growth restriction is marked. The adjusted mean growth deficit with gastroschisis was 317 g. This growth deficit could not be explained by maternal factors, which might be common to mothers delivering a baby with either gastroschisis or with growth restriction associated with another condition [17,18]. Our limited prenatal ultrasound data suggested that growth restriction began early in the second trimester and worsened with increasing gestation. This is consistent with others' observations [10-13]. Taken together our data confirm that intrauterine growth restriction is a near universal finding with a gastroschisis defect.

The degree of intrauterine growth restriction was actually greater than that seen with chromosomal anomalies. The ponderal index was very slightly increased, consistent with a reduction in length that was proportional to the reduction in weight. The relatively symmetrical nature of the growth restriction probably reflects early onset [35]. Compounding intrauterine growth deficits, GI dysfunction contributes to suboptimal nutrition after birth in the neonatal period [5,6,36].

Our findings imply that gastroschisis patients, because of their marked intrauterine growth restriction, are at increased risk for cardiovascular disease, adult-onset diabetes, and perhaps most importantly, intellectual disability [37,38]. Leonard, et al., found that growth restriction increased the odds of intellectual disability by about 71% in a large Australian cohort [38]. Most gastroschisis patients are born in the late preterm period (mean gestational age at birth = 36 weeks). Late preterm birth has also been associated with adverse cognitive and socioemotional outcomes [39]. Gastroschisis patients warrant close monitoring for developmental and intellectual problems throughout childhood [40,41].

Our study extends pathogenetic considerations to include the placenta. Placental histology suggested that placental dysfunction may contribute to growth restriction. Chorangiosis and severe villous edema were more common in gastroschisis patients than in controls. Chorangiosis is an increase in vascular channels in the terminal villi of the placenta. It is thought to represent fetal hypoxemia and the placenta's attempt to improve gas exchange across the terminal villi [31] and takes weeks to develop. It has been associated with delivery at high altitude, severe maternal anemia, and diabetes mellitus [31,32]. Villous edema also suggested placental dysfunction. In a separate study, we found evidence that gestational hypertension is less common in the mothers of gastroschisis patients [42]. The placenta plays a central role in the development of gestational hypertension [43]. It is unclear how the development of gastroschisis in the fetus might be associated with decreased gestational hypertension in the mother. The placenta is often small in cases of gestational hypertension associated with fetal growth restriction [43]. However, we did not find a significant difference in the placental weight z-score between gastroschisis patients and controls. Stoll, et al. also reported that placental size was not reduced in a smaller series of gastroschisis cases [44]. These findings, if confirmed, suggest that the placenta may develop abnormally and lead to growth restriction in gastroschisis cases, but not in the manner seen with gestational hypertension.

The exact mechanism by which growth restriction occurs in gastroschisis cases is not known. Carroll, et al. reproted diminished cord serum protein and elevated amniotic fluid protein in the amniotic fluid compared to omphalocele patients and controls [45]. Unfortunately, the patients and controls were not well matched with respect to gestational age, which could have affected the results. Protein loss through exudation of proteinaceous fluid from the intestine, which is often inflamed and exposed to the amniotic fluid throughout gestation, might well contribute to poor intrauterine growth, but probably would not account for chorangiosis. Chorangiosis provided indirect evidence of poor oxygen transfer from the placenta to the fetus. The presence of intestinal obstruction or dysfunction did not likely contribute to fetal growth restriction, since other GI anomalies, most of which were atresias, were not associated with decreased birth weight. It is possible that multiple mechanisms contribute to poor fetal growth in the presence of gastroschisis.

Several limitations should be considered when interpreting our study. All cases were referred and do not represent population-based data. However, the demographic findings of our patients are similar to previous reports [2-6]. Another concern might be error in physical measurements obtained by clinical personnel. BW of gastroschisis patients could have been biased upward due to bowel edema or the bandages used to protect exposed intestine. However, nurses routinely weigh and subtract the weight of bandages when recording BW. Even if present, this bias would have reduced the observed BW difference between cases and controls and cannot explain our findings. Furthermore, measurements of LT and OFC, were also low and not as likely to have been influenced by the presence of gastroschisis. We were limited in the number of prenatal ultrasounds available for study. Patients were often evaluated and received their prenatal ultrasounds at satellite clinics. Only when patients received their prenatal ultrasound at the main perinatal center were the ultrasound data available to us. The strengths of this study are the large numbers of patients examined, the multiple approaches to quantifying the growth deficit and our examination of placental findings.

## Conclusion

We report that relatively symmetric, intrauterine growth restriction occurs in almost all gastroschisis patients to some degree. These growth deficits are comparable to those seen in chromosomal disorders and are associated with decreased length and head circumference. Growth deficits increased from early in the second trimester until delivery, and in many cases continued through neonatal period. We found chorangiosis, a response to tissue hypoxia, to be more common in gastroschisis patients than in controls, which may implicate a placental contribution to growth restriction.

## Abbreviations

BW: birth weight; GI: gastrointestinal; g: grams; LT: crown-heel length; NICU: newborn intensive care unit; OFC: occipitofrontal circumference; PI: ponderal index; sd: standard deviation; SGA: small for gestational age

## Competing interests

The authors declare that they have no competing interests.

## Authors' contributions

NRP conceived, planned and organized the study. He also wrote the manuscript. SCS reviewed all of the placental slides. SO collected, collated and partially analyzed clinical data. MAA made available and reveiwed placental slides. KMP made available all prenatal ultrasound data and participated in the analysis of the ultrasound data. All authors reviewed the manuscript and approved its content.

## Pre-publication history

The pre-publication history for this paper can be accessed here:

http://www.biomedcentral.com/1471-2431/11/90/prepub
